# Lower Limb Amputation Rates in Germany

**DOI:** 10.3390/medicina58010101

**Published:** 2022-01-10

**Authors:** Nike Walter, Volker Alt, Markus Rupp

**Affiliations:** 1 Department for Trauma Surgery, University Hospital, 93053 Regensburg, Germany; nike.walter@ukr.de (N.W.); volker.alt@ukr.de (V.A.); 2 Department for Psychosomatic Medicine, University Hospital, 93053 Regensburg, Germany

**Keywords:** amputation, lower extremity, epidemiology, diabetes mellitus, peripheral arterial disease

## Abstract

*Background and Objectives*: The current epidemiology of lower limb amputations is unknown. Therefore, the purpose of this study was to determine (1) lower extremity amputation rates as a function of age, gender, and amputation level between 2015 and 2019, (2) main diagnoses indicating amputation, (3) revision rates after lower extremity amputation. *Materials and Methods*: Lower extremity amputation rates were quantified based on annual Operation and Procedure Classification System (OPS) and International Classifications of Disease (ICD)-10 codes from all German medical institutions between 2015 through 2019, provided by the Federal Statistical Office of Germany (Destatis). *Results*: In 2019, 62,016 performed amputations were registered in Germany. Out of these 16,452 procedures (26.5%) were major amputations and 45,564 patients (73.5%) underwent minor amputations. Compared to 2015, the incidence of major amputations decreased by 7.3% to 24.2/100,000 inhabitants, whereas the incidence of minor amputation increased by 11.8% to 67.1/100,000 inhabitants. Highest incidence was found for male patients aged 80–89 years. Patients were mainly diagnosed with peripheral arterial disease (50.7% for major and 35.7% for minor amputations) and diabetes mellitus (18.5% for major and 44.2% for minor amputations). *Conclusions*: Lower limb amputations remain a serious problem. Further efforts in terms of multidisciplinary team approaches and patient optimization strategies are required to reduce lower limb amputation rates.

## 1. Introduction

Lower limb amputations are usually performed to excise necrotic tissue and can have multiple causes such as diabetes mellitus (DM), peripheral arterial disease (PAD), bone and joint infections, peripheral neuropathy, trauma, or malignancy [1]. Especially, diabetic foot complications are the leading reason for non-traumatic lower extremity amputations [2,3]. In the Saint Vincent Declaration of 1989, it was targeted that amputation rates due to diabetes should be halved within 5 years [4]. However, the global prevalence of diabetes mellitus is increasing. According to projections, a burden up to 7079 individuals per 100,000 inhabitants can be forecasted by 2030 [5]. Additionally, cases of PAD have been heightening, likely due to the demographic trend of aging [6]. Both diagnoses increase the risk for amputations [7].

Despite these increases, the incidence of major amputations declined in Europe [8,9,10] as well as in the U.S. [11]. For instance, a decrease from 27.5 to 25.0 per 100,000 persons with DM and a decrease from 13.6 to 11.9 per 100,000 persons without DM was reported for the U.K. between 2004 and 2008 [12]. In Italy, during 2001–2010 the amputation rate decreased to 20.4 per 100,000 inhabitants [8], whereas in Belgium an 8% annual reduction was observed from 2009 to 2013 to 29.9 per 100,000 person-years for patients with DM [13]. An analysis of national hospital discharge data from Finland between 1997 and 2007 revealed a decrease from 13.6 to 9.3 per 100,000 person-years for people with DM [14]. Actual reliable data based on nationwide registries is important for stakeholders in health care systems in order to estimate future demands and evaluate advances in diabetic care and vascular medicine [15]. However, studies quantifying amputation rates only considered population-based data with latest updates for the year 2015 and the current epidemiology of lower limb amputations is unknown for European countries.

Therefore, we aimed to determine (1) lower extremity amputation rates divided by age, sex, and amputation level between 2015 and 2019, (2) main diagnoses indicating amputation, (3) revision rates after lower extremity amputation.

## 2. Materials and Methods

For this cross-sectional study, amputation data from 2009 to 2019 was provided by the Federal Statistical Office of Germany (Destatis). The data consisted of annually reported surgical procedures performed in medical institutions of all 16 German federal states. The data included all inpatient procedures. The coding is usually performed by physicians. Surgery and procedure keys (Operation and Procedure Classification System codes) were used to identify all performed lower extremity amputations in patients aged 20 years or older, regardless of the underlying disease or injury in the years 2015 and 2019. In particular, the Operation and Procedure Classification System (OPS) codes “5-864, lower limb amputation and disarticulation”, and “5-865, foot amputation and disarticulation” were used. OPS codes were grouped regarding the amputation level in major amputations including hemipelvectomy, hip disarticulation, transfemoral amputation, knee disarticulation, transtibial amputation, and unspecified lower extremity amputation as well as in minor amputations consisting of transmalleolar amputation (Syme technique), foot amputation, partial food amputation, and toe amputation (Table 1). For the determination of revision rates the OPS codes “5-866.3, Revision of femoral amputation site”, “5-866.4, Revision of lower leg amputation site” and “5-866.5, Revision of foot amputation site” were used. A detailed breakdown of these data by age group in 10-year increments and sex was performed for each amputation level, respectively.

For the analysis of the underlying main diagnoses, these were grouped into peripheral arterial disease (PAD) (ICD-10 code I7), diabetes mellitus (DM) (ICD-10 codes E10-E14), and a category termed “others” including the principal diagnosis of tumor (ICD-10 codes C00-D48), cutaneous or subcutaneous disease (ICD-10 codes L00-L99), trauma (ICD-10 codes S72, S82, S88, S92, T02, T12), musculoskeletal disease (ICD-10 codes M0-M9), sepsis (ICD-10 codes A40, A41), and complication due to prosthesis, implant, or transplant (ICD-10 codes T82, T84).

Data were analyzed using the statistical software SPSS Version 26.0 (IMB, SPSS Inc. Armonk, NY, USA). Categorical data is expressed as frequency counts (percentage). Incidence rates were calculated based on Germany population aged 20 years or older for the years 2015 and 2019, respectively, provided by Destatis. Here, the number of inhabitants in each of the 16 German federal states was considered by year of birth for each year. The deadline of each year was December 31. Incidence rates were adjusted for age and sex. Incidence rate ratios and the according 95% confidence interval were calculated.

This is a purely observational study. The Research Ethics Committee of the University Hospital Regensburg has confirmed that no approval and no informed consent is required. The study was performed in accordance with the ethical standards in the 1964 Declaration of Helsinki. No administrative permission was required to access the analyzed dataset. The data was anonymized before its use.

## 3. Results

**Figure 1:**
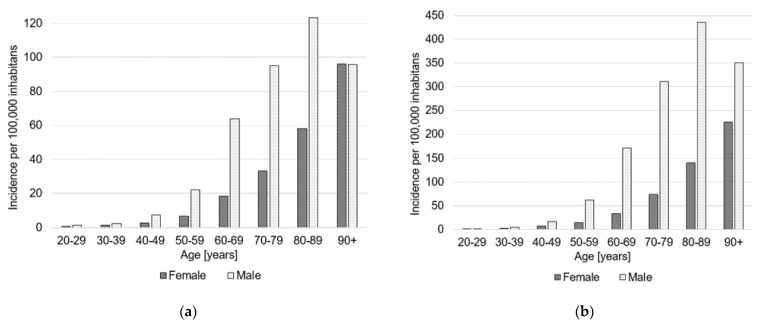
Age and sex adjusted incidence rates per 100,000 inhabitants for the year 2019. (**a**) Major amputations, (**b**) minor amputations. Female cases are shown in dark grey and male cases in light grey.

**Figure 2:**
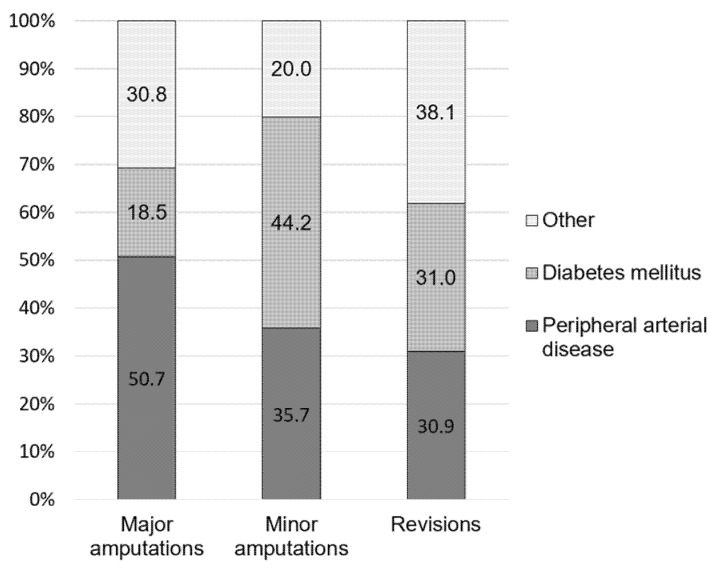
Main diagnoses of patients undergoing major amputations, minor amputations, and revision surgeries of an amputation site in 2019.

In 2019, a total number of 62,016 performed amputations were registered in Germany. Out of these 16,452 procedures (26.5%) were major amputations and 45,564 patients (73.5%) underwent minor amputations. In comparison to 2015, the incidence of major amputations decreased by 7.3% from 26.2 per 100,000 inhabitants to 24.2 per 100,000 inhabitants (Table 2). Men were more often affected than women (68% vs. 32%) and the majority of patients was older than 70 years (58%). The incidence rate steadily increased with age, with the highest incidence (95.9 per 100,000 inhabitants) for patients older than 90 years (Table 3, Figure 1a). The most common major amputation was the transfemoral amputation with an incidence of 13.3 per 100,000 inhabitants, whereby procedures increased by 71% since 2015. This was followed by transtibial amputations which increased by +7% over the years to an annual number of 6455 procedures in 2019 (Table 2). The majority of patients who underwent major amputations was diagnosed with PAD (50.7%), whereas 18.5% were diagnosed with DM (Figure 2).

The incidence of minor amputation increased by 11.8% from 60.1 per 100,000 inhabitants to 67.1 per 100,000 inhabitants and 45,564 annual procedures in 2019 (Table 2). Additionally, here more amputations were performed on men than women (74% vs. 26%) with most patients being older than 70 years (62%). The age and sex distribution showed a similar picture for the major amputations. The incidence increased steadily with age in the female population with a maximum of 225 per 100,000 inhabitants in the age group older than 90 years. The highest incidence in the male population was found for the age group 80–89 years old (435 per 100,000 inhabitants) (Table 3, Figure 1b). Most frequently performed procedures in 2019 included toe amputations (35,995) and partial foot amputations (9189) (Table 2). The majority of patients who underwent minor amputations was diagnosed with DM (44.2%), whereas 35.7% were diagnosed with PAD (Figure 2).

In 2019, 8833 revision procedures of an amputation site were performed. In comparison to 8071 revision procedures in 2015, numbers rose by 8.6%. Foot amputation site revisions were most prevalent with 5785 procedures, whereby the revision rate was highest for the lower leg (0.24). Additionally, the latter showed the highest increase in comparison to 2015 (+14.5%). Here as well, men required more revision surgeries than women, whereby the incidence for patients aged older than 70 years was elevated (Table 4). Indications for revision surgery of the amputation site was DM in 31% of the cases and PAD in 30.9% of the cases (Figure 2).

## 4. Discussion

In this population-based study the epidemiology of lower limb amputations was described, and incidences were analyzed as a function of age, sex, and amputation level. Whereas some studies provided insights regarding the trends of amputation rates [8,9,10,11], to the best of our knowledge, the nationwide burden of lower limb amputations in Germany has only been reported dating back until the year 2015.

In comparison to 2015, the incidence of major amputations decreased by 7.3% to 24.2 per 100,000 inhabitants, whereas the incidence of minor amputation increased by 11.8% to 67.1 per 100,000 inhabitants. The finding that major amputations decreased, whereas minor amputations increased is in line with German data from previous years [9,16,17]. For instance, Santosa et al. used data provided by Destatis to determine amputation rates in Germany for the years 2005–2010 estimating a decrease of 4.8/100,000 inhabitants for major amputations and an increase of 5.5/100,000 inhabitants for minor amputations [16]. Additionally, Kröger and colleagues analyzed lower limb amputation in Germany from 2005 through 2014, reporting a decrease of major amputations of 30.9% and an increase of minor amputations of 25.4% [9]. Further, the in-hospital mortality of cases with lower limb amputations was shown to decline from 11.2% to 7.7% between 2005 and 2015 [17]. The results further revealed that men who underwent major as well as minor amputations were more often with a maximum incidence in the age group 80–89 years old. For females, amputation rates increased with age with highest incidences in the age group 90+ years old. This is in line with other findings reporting increasing amputation rates with age and male gender as well as a higher risk for foot ulceration for male patients [18,19,20]. In addition, revision amputation procedures rose by 8.6% with highest revision rates for the lower leg (0.24). Another study including 2879 patients reported that 41% required at least one revision amputation [21]. Additionally, re-amputation rates of 20.14% at 1 year were reported for patients with initial diabetic foot ulcers [2]. Here, 50.7% of the patients who underwent major amputations were diagnosed with PAD. This in line with other findings, showing that PAD increased the risk of lower limb amputations four-fold in centricity electronic medical records from the U.S. [22]. Therefore, PAD management policies were proposed to effectively reduce the rate of nontraumatic lower limb amputation [23]. For minor amputation, most patients were diagnosed with DM. A recent meta-analysis including literature until December 2014 reported that the incidence of lower limb amputations in the diabetic population ranged from 78 to 704 per 100,000 person-years and that the relative risks between diabetic and non-diabetic patients varied between 7.4 and 41.3 [24]. Using data from Belgian national health insurance fund, Claessen and colleagues observed a significant decline in major amputation rates among people with diabetes, whereas the major amputation rate remained stable in patients without diabetes [13]. In contrast, in the U.S. an increase of both major and minor amputations was observed among patients with diabetes from 2009 to 2015 [25]. In this stance, it was further investigated whether the choice of anti-diabetic drugs influences amputation rates amongst patients with type 2 diabetes mellitus. Here, it was shown that lower limb amputation rates were similar between patients treated with a new class of anti-diabetic therapy, sodium-flucose cotransporter type-2 inhibitors compared to others [22]. However, it was also suggested that canagliflozin might be positively associated with an increased risk of amputation [26]. In addition, different aspects potentially contributing to less major amputation rates were investigated. It was found that the prescription of podologic foot care was a significant factor and that the utilization of podologic foot care was strongly associated with decreasing major amputations in Germany [9,27].

Recently, it has been emphasized that uniform definitions of the amputation level are required to ensure international comparability. In this stance, it was highlighted that analyses based on German diagnosis-related groups (DRG) may be challenging as for instance the Syme procedure is defined as a minor amputation [15]. Therefore, in this study OPS-codes were used to quantify incidences for distinct amputation levels. Notably, in this study a decrease of 95% was found for unspecified amputations, which might reflect advances in coding over the years. This aspect might directly impact the reported increase in other amputation procedures such as transfemoral amputations and has to be taken into account as a limitation regarding generalization of the findings. Additionally, analyses based on registry data show several limitations. The main one comprises the unverifiable accuracy of coding and data input. Thus, potential misclassification or a possible upcoding cannot be excluded. However, accurate coding is assumable since DRG lump sum reimbursement relies on it and is strictly controlled by the Medical Service of Health Funds. Nevertheless, future validation studies of the OPS codes should be considered. Further, the structure of the data only allowed a purely descriptive study design. In addition, the underlying principal diagnoses have to be interpreted with caution as it cannot be ensured that the amputations were primarily related to diabetes mellitus or peripheral arterial disease. Further, due to the data structure, it was not possible to derive individual patient medication. In the same stance, even though underlying main diagnoses were available in total numbers, these could not be provided specifically divided by age and sex. Finally, the dataset only includes stationary data. However, as amputation is an inpatient procedure potential biases can be regarded as small.

## 5. Conclusions

Lower limb amputations remain a serious problem. Further efforts in terms of multidisciplinary team approaches and patient optimization strategies are required to reduce lower limb amputation rates.

## Figures and Tables

**Table 1:** Operation and Procedure Classification System code descriptions.

Operation and Procedure Classification System Code	Description
5-864.0 + 5-864.1	Hemipelvectomy
5-864.2	Hip disarticulation
5-864.3 + 5-864.4 + 5-864.5	Transfemoral amputation (above-the-knee)
5-864.6 + 5-864.7	Knee disarticulation
5-864.8 + 5-864.9 + 5-864.a	Transtibial amputation
5-864.x + 5-864.y	Lower extremity unspecified
5-865.0	Transmalleolar amputation
5-865.1 + 5-865.2 + 5-865.3	Foot amputation
5-865.4 + 5-865.5 + 5-854.6	Partial food amputation
5-865.7 + 5-865.8	Toe amputation

**Table 2:** Incidence rates of the distinct amputation procedures performed in the year 2019 compared to 2015.

Anatomical Localization	Total Numbers		Incidence per 100,000 Inhabitants		Incidence in 2019 Relative to 2015 (%)	Incidence Rate Ratio [95% CI]
	2019	2015	2019	2015		
Major amputations	16,452	17,546	24.2	26.1	−7.3%	0.93 [0.91–0.95]
Hemipelvectomy	63	28	0.1	0.0	+122.5%	2.22 [1.32–3.76]
Hip disarticulation	154	109	0.2	0.2	+39.7%	1.40 [1.07–1.82]
Transfemoral amputation	9015	5201	13.3	7.8	+71.4%	1.71 [1.65–1.78]
Knee disarticulation	700	4972	1.0	7.4	−86.1%	0.14 [0.13–0.15]
Transtibial amputation	6455	5963	9.5	8.9	+7.0%	1.07 [1.03–1.11]
Lower extremity unspecified	65	1273	0.1	1.9	−95.0%	0.05 [0.04–0.06]
Minor amputations	45,564	40,308	67.1	60.1	+11.8%	1.12 [1.10–1.13]
Transmalleolar amputation	157	47	0.2	0.1	+230.3%	3.30 [2.20–4.95]
Foot amputation	223	346	0.3	0.5	−36.3%	0.64 [0.55–0.74]
Partial foot amputation	9189	5166	13.5	7.7	+75.9%	1.76 [1.69–1.82]
Toe amputation	35,995	34,749	53.0	51.8	+2.4%	1.02 [1.01–1.04]

**Table 3:** Age-adjusted incidence rate of major and minor amputations in the years 2015 and 2019, respectively. IRR = incidence rate ratio.

	20–29 Years (Males, Females, Total)	30–39 Years (Males, Females, Total)	40–49 Years (Males, Females, Total)	50–59 Years (Males, Females, Total)	60–69 Years (Males, Females, Total)	70–79 Years (Males, Females, Total)	80–89 Years (Males, Females, Total)	90 Years or Older (Males, Females, Total)	All (Males, Females, Total)
Major amputations in 2015	1.2, 0.2, 0.7	2.1, 0.8, 1.5	5.8., 3.4, 4.6	24.3, 8.1, 16.2	65.2, 19.3, 41.5	102.5, 37.5, 67.0	148.0, 82.3, 106.4	103.7, 142.1, 130.5	34.6, 18.1, 26.1
Major amputations in 2019	1.2, 0.5, 0.9	2.3, 1.4, 1.9	7.2, 2.7, 4.9	22.2, 6.7, 14.4	63.9, 18.4, 40.4	95.0, 33.1, 61.4	123.3, 58.0, 83.4	95.6, 96.0, 95.9	33.8, 15.2, 24.3
IRR major amputations	0.98, 2.16, 1.16	1.07, 1.70, 1.25	1.24, 0.78, 1.07	0.92, 0.83, 0.89	0.98, 0.95, 0.98	0.93, 0.88, 0.92	0.83, 0.71, 0.78	0.92, 0.68, 0.73	0.98, 0.84, 0.93
Minor amputations in 2015	1.8, 0.6, 1.2	4.2, 2.1, 3.1	14.6, 5.6, 10.2	53.5, 14.8, 34.2	149.6, 34.4, 90.1	271.0, 82.5, 168.0	389.0, 169.9, 250.3	265.3, 241.5, 248.7	85.4, 35.9, 60,1
Minor amputations in 2019	1.5, 1.1, 1.3	4.7, 1.9, 3.4	17.0, 6.4, 11.7	61.0, 13.9, 37.4	171.1, 33.3, 100.1	310.8, 74.0, 182.3	435.2, 140.1, 254.9	350.4, 225.0, 266.7	102.5, 33.5, 67.2
IRR minor amputations	0.97, 1.81, 1.10	1.14, 0.94, 1.07	1.16, 1.15, 1.16	1.14, 0.94, 1.09	1.14, 0.97, 1.11	1.15, 0.90, 1.09	1.12, 0.82, 1.02	1.32, 0.93, 1.07	1.20, 0.93, 1.12

**Table 4:** Incidence rates of revision amputation procedures performed in the year 2019 divided by anatomical localization.

Anatomical Localization	Total Numbers	Revision Rate	Revision Rate 2019 Relative to 2015 (%)	Percentage Female/Male	Incidence Female/Male	Percentage Aged ≤70 Years/>70 Years	Incidence Aged ≤70 Years/>70 Years
Femur	1502	0.16	+11.8%	38/62	1.7/2.8	56/44	1.5/5.0
Lower leg	1546	0.24	+14.5%	25/75	1.1/3.5	61/39	1.7/4.5
Foot	5785	0.13	+1.3%	20/80	3.3/14.0	44/56	4.6/24.7

## Data Availability

The datasets analyzed during the current study are available from the corresponding author on reasonable request.
